# Combined Rapid (TUBEX) Test for Typhoid-Paratyphoid A Fever Based on Strong Anti-O12 Response: Design and Critical Assessment of Sensitivity

**DOI:** 10.1371/journal.pone.0024743

**Published:** 2011-09-15

**Authors:** Meiying Yan, Frankie C. H. Tam, Biao Kan, Pak Leong Lim

**Affiliations:** 1 State Key Laboratory for Infectious Disease Prevention and Control, National Institute for Communicable Disease Control and Prevention, Chinese Center for Disease Control and Prevention, Beijing, People's Republic of China; 2 IgGENE, Fotan, Hong Kong; Charité-University Medicine Berlin, Germany

## Abstract

Rapid diagnostics can be accurate but, often, those based on antibody detection for infectious diseases are unwittingly underrated for various reasons. Herein, we described the development of a combined rapid test for two clinically-indistinguishable bacterial diseases, typhoid and paratyphoid A fever, the latter fast emerging as a global threat. By using monoclonal antibodies (mAbs) to bacterial antigens of known chemical structures as probes, we were able to dissect the antibody response in patients at the level of monosaccharides. Thus, a mAb specific for a common lipopolysaccharide antigen (O12) found in both the causative organisms was employed to semi-quantify the amounts of anti-O12 antibodies present in both types of patients in an epitope-inhibition particle-based (TUBEX) immunoassay. This colorimetric assay detected not only anti-O12 antibodies that were abundantly produced, but also, by steric hindrance, antibodies to an adjoining epitope (O9 or O2 in the typhoid or paratyphoid bacillus, respectively). Sensitivity and, particularly, reaction intensities, were significantly better than those obtained using an anti-O9 or anti-O2 mAb-probe in the examination of paired sera from 22 culture-confirmed typhoid patients (sensitivity, 81.8% vs 75.0%) or single sera from 36 culture-confirmed paratyphoid patients (52.8% vs 28.6), respectively. Importantly, sensitivity was better (97.1% for typhoid, 75.0% for paratyphoid) if allowance was made for the absence of relevant antibodies in certain specimens as determined by an independent, objective assay (ELISA) — such specimens might have been storage-denatured (especially the older paratyphoid samples) or procured from non-responders. Benchmarking against ELISA, which revealed high concordance between the two tests, was useful and more appropriate than comparing with culture methods as traditionally done, since antibody tests and culture target slightly different stages of these diseases. Paired sera analysis was insightful, revealing 64% of typhoid patients who had no change in antibody titer over 4–16 days, and 14% with no IgM-IgG class-switching.

## Introduction

Sensitivity and specificity characterize how clinically useful an immunodiagnostic (serological) test is. For acute infectious diseases, these parameters are usually computed by comparing the results obtained from such a test with those derived by culture of the infectious agent from the study cohort. This time-honored use of culture as the gold standard in diagnosis has seldom been questioned even though in many situations, culturing is neither sensitive nor practicable [1], [2], [3]. This raises the possibility that, in some cases, the real worth of immunodiagnostic tests could be unfairly benchmarked. Another factor often taken for granted which, too, can adversely affect the perceived performance of immunodiagnostic tests is the quality of the specimen used for analysis, particularly if, as is often done, the specimen has been procured years ago and stored away.

We addressed the above issues in the present study using typhoid fever as the model disease. This is historically appropriate because the first immunodiagnostic test in the world—the Widal test [4]—was developed for typhoid, a test that is still widely used today. Typhoid has remained a major health threat globally, affecting some 20 million people annually [5]. A related disease, paratyphoid fever A, which resembles typhoid fever clinically and which is probably under-diagnosed, has recently emerged to be as dangerous as typhoid fever [6]. Study of both diseases together allows assay sensitivity and specificity to be addressed more comprehensively, while the availability of detailed information regarding both of the infecting organisms, including, in particular, the relevant antigens, permits high-resolution analysis of specificity.

Thus, the organisms that cause typhoid and paratyphoid A fever, *Salmonella enterica* serotype Typhi (*S*. Typhi) and *S*. Paratyphi A, respectively, belong to a large family of *Salmonella* organisms. There are about 2,000 members or serotypes differentiated by the surface “O” and “H” antigens found in the lipopolysaccharide (LPS) and flagella, respectively, of these bacteria. Serotypes with a common immunodominant “O” antigen are grouped together [7]. Thus, *S*. Typhi belongs to serogroup D in which the common antigens are O9 and O12, while *S*. Paratyphi A belongs to serogroup A which has O2 and O12. Another member which causes paratyphoid fever but found infrequently and only in certain geographical regions, *S*. Paratyphi B, belongs to serogroup B (O4 and O12). A third paratyphoid member, *S*. Paratyphi C, which is also infrequently found and causes a milder disease, belongs to serogroup C (O6 and O7). All other serotypes of *Salmonella* are usually not invasive and cause a less debilitating local infection in the gut.

An understanding of the pathogenesis of typhoid fever is germane to the appreciation of assay sensitivity and specificity. Thus, disease is initiated following ingestion of *S*. Typhi organisms, which then multiply in the mesenteric tissues before disseminating into the bloodstream and seeding the various organs, all taking a week or two usually [8]. At this early stage, the organism is readily recovered from the circulation while antibody production to the organism may have only just begun, starting with IgM antibodies. As the disease progresses into late phase and convalescence after, usually, 3–4 weeks, the organism circulates only infrequently in the blood while antibodies, on the other hand, become increasingly abundant. As the disease terminates, the organism disappears completely from the host and pathogen-specific IgM antibodies also subside steadily in levels, but IgG antibodies can remain elevated for months thereafter. However, in about 5% of typhoid patients who become chronic carriers following remission, the organism hides away in the gall bladder and persists indefinitely.

Detailed chemical structures of the “O” antigens have long been known [9]. Importantly, many of the antigens are specified by only a few sugar molecules. This allows precise analysis of specificities to be made at the level of monosaccharides. For example, O9 is specified largely by an unusual sugar located at the end of a repeating oligosaccharide chain in the LPS. This is tyvelose, a dideoxymannose, which adjoins a trisaccharide in the chain (mannose-rhamnose-galactose) that specifies the O12 antigen. O12 is also present in *S*. Paratyphi A and *S*. Paratyphi B, but in these organisms, O9 is substituted by O2 (paratose) and O4 (abequose), respectively. Both paratose and abequose are structurally identical to tyvelose except for the stereoisomeric orientation of an –OH group (Steinbacher, 1996). Uniquely, these dideoxyhexoses (LPS as a whole) are thymus-independent type 1 antigens which stimulate strong and rapid IgM antibody responses even in infants [10].

The Widal test is the first to exploit the unique attributes of the “O” antigens to detect serum antibodies from typhoid patients, but because whole bacterial cells are employed in agglutination tests, this test is neither sensitive nor specific [11], [12]. Better performance is observed when purified LPS extracted from *S*. Typhi organisms is used in ELISA tests [13]. We exploit the high specificity of these antigens in a more precise manner, by utilizing monoclonal antibodies (mAbs) to the various “O” antigens in conjunction with whole LPS in epitope-inhibition assays. Thus, anti-O9 antibodies in patients are detected by their ability to inhibit the binding between an anti-O9 mAb and the O9 antigen in the LPS. For easy and rapid visualization, the mAb is coupled to blue-colored indicator particles, and the LPS to magnetic particles. Following brief and vigorous agitation in V-shaped microwells, the reaction mixture comprising test serum and reagent particles is placed on a magnet stand to sediment the magnetic particles. Indicator particles bound to these will be co-sedimented. Thus, presence of infection-specific antibodies will be revealed by the resultant concentration (color) of indicator particles left in the suspension. A background color (red) is added so that results are visually and semi-quantitatively read based on varying mixtures of blue and red.

TUBEX TF® (IDL Biotech, Bromma, Sweden) is the first application of the above diagnostic system. It uses an anti-O9 mAb (3h1) to detect typhoid fever, and results are scored against a color chart with a scale of 0 to 10 (score ‘0’, negative and most red; score ‘10’, most positive and most blue). Reasonable sensitivities (75–90%) and specificities (70–97%) have been observed [12]–[17]. More recently, we developed a prototypic test (TUBEX PA) for paratyphoid A fever which employed an anti-O2 mAb (P1D10) and *S*. Paratyphi A LPS, which also yielded reasonable sensitivities (81–93%) [18]. However, a surprising finding from this study was that TUBEX PA could also detect typhoid patients in about 50% of cases, and the converse was true with TUBEX TF in regard to paratyphoid A patients. We reasoned that this mutual cross-detection was due to the presence of anti-O12 antibodies in both cases, the possibility being that these antibodies could interfere in the test by steric hindrance due to the close proximity of the O12 and O9 or O2 antigens.

In the present study, we exploited the presence of anti-O12 antibodies in both typhoid and paratyphoid A patients to establish a combined TUBEX test for these diseases. An anti-O12 mAb was thus employed, which impressively, proved to be a highly sensitive indicator. Sensitivity is attributed to the abundance of anti-O12 antibodies in these patients, which appeared to consist of at least 2 sub-populations, as well as the fact that anti-O9 and anti-O2 antibodies are also detected in the combined test by virtue of steric hindrance. An important revelation is that the type or quality of specimen used can seriously affect the sensitivity of the assay. We found inclusion of an independent and objective test such as ELISA to be extremely important since this can serve both as a check on specimen quality and as a performance yardstick for the rapid test.

## Results

### Preliminary studies show O12 indicator more sensitive than O9 or O2 indicator

**Figure 1:**
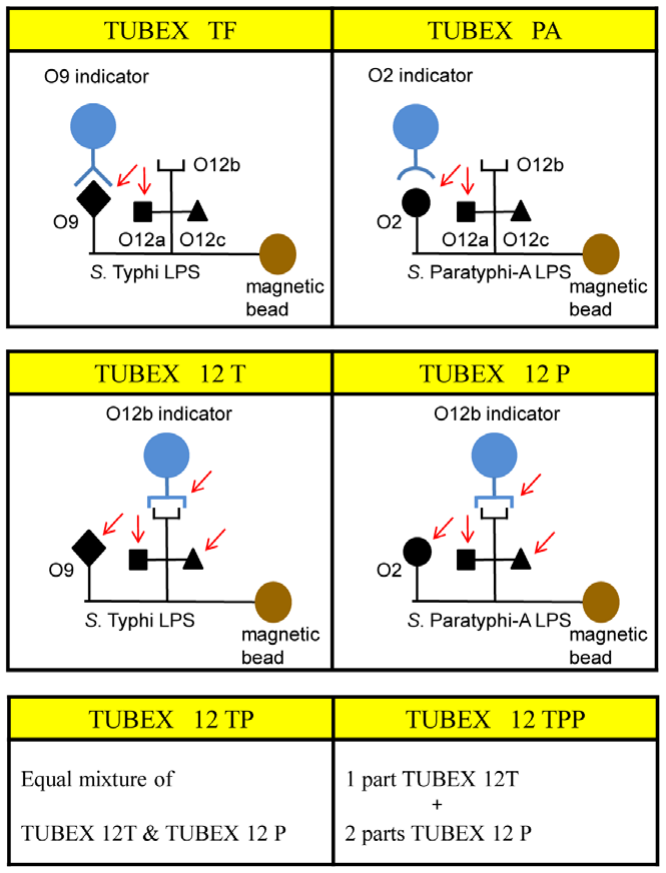
Schematic diagram illustrating the various types of TUBEX tests and the various types of antibodies detected by these. Shown are the various mAb-indicators (anti-O9, anti-O2 or anti-O12b) and antigenic epitopes (O9, O2 O12a, O12b and O12c) found in *S*. Typhi or *S*. Paratyphi A LPS. Arrow indicates antigenic epitope that antibodies from patients presumptively bind to and inhibit binding of the indicator particle.

**Figure 2:**
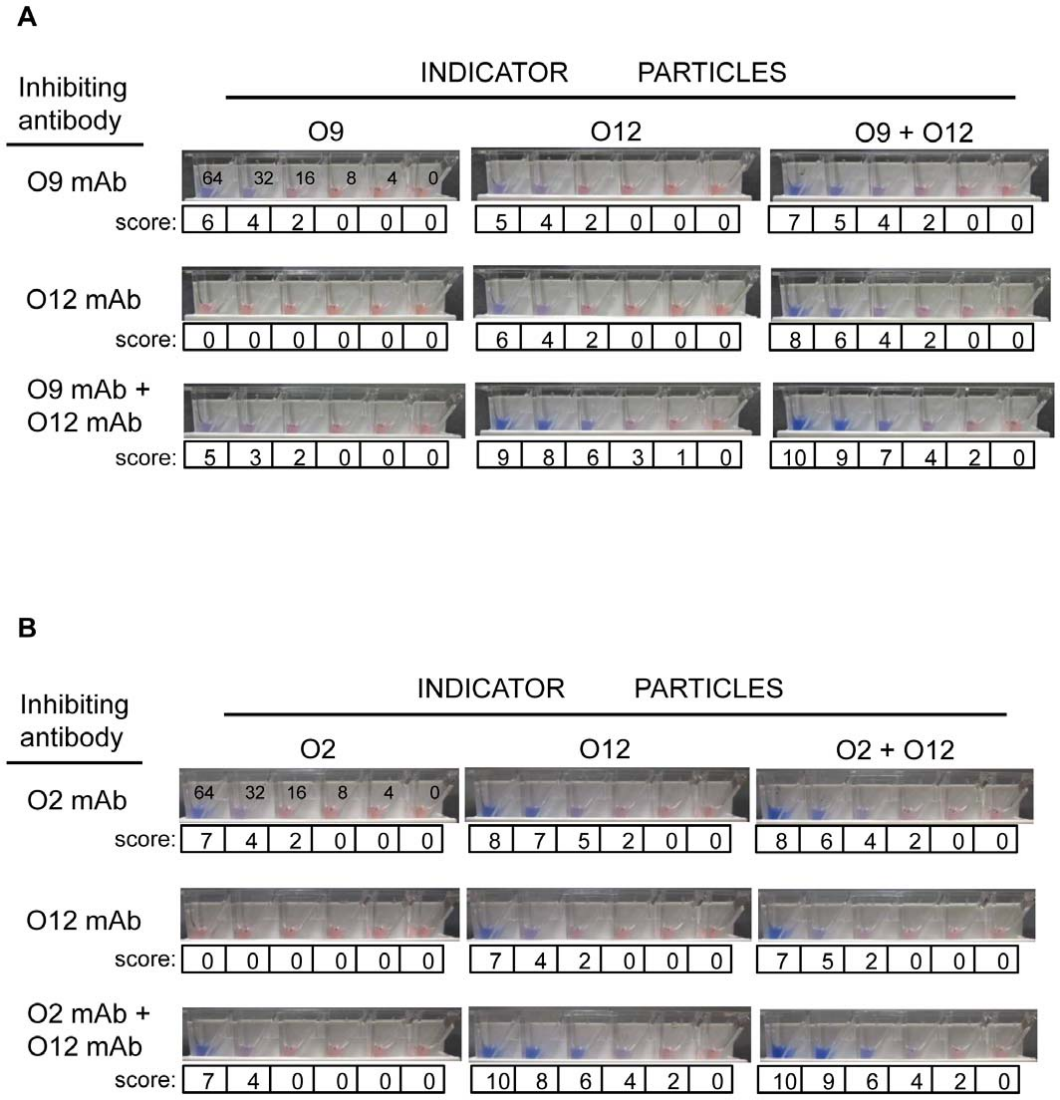
Pictoral results showing efficacy of various TUBEX tests in detecting mAb-spiked test samples. Various mAb-indicators (anti-O9, anti-O2 or anti-O12b) in conjunction with O9 LPS- (A) or O2 LPS-coupled (B) magnetic particles to detect various types of purified mAbs (anti-O9, anti-O2 or anti-O12b, or combinations of these). Numericals in microwells denote individual mAb concentration (µg/ml), same for all sets of microwells; ‘score’ denotes TUBEX results.

We prepared blue latex particles coupled with the anti-O12 mAb (P4E8) and examined the performance of these as substitute indicator particles in various TUBEX tests (see Fig. 1). First, we compared the sensitivity of detection of purified mAbs by this O12 indicator (TUBEX 12T) with the O9 indicator (also blue-colored; TUBEX TF), both situations using *S*. Typhi LPS as substrate. It is evident that the O12 indicator detected the anti-O9 mAb (3h1) as efficiently (32 µg/ml) as the O9 indicator (Fig. 2A). While the O12 indicator also detected the anti-O12 mAb (P4E8) at a similar sensitivity, interestingly, the O9 indicator was not able to detect this mAb. When both inhibiting mAbs were used together, the O9 indicator detected this combination no better than detecting 3h1 alone; in contrast, increased sensitivity (16 µg/ml) and increased intensity of reaction were observed using the O12 indicator. When both the O12 and O9 indicators were used together in the test, results similar to or marginally better than those using the O12 indicator alone were obtained (Fig. 2A).

Second, we compared the performance of the O12 indicator (TUBEX 12P) with the O2 indicator (coupled with mAb P1D10; TUBEX PA) both using *S*. Paratyphoid A LPS, to detect purified mAbs (anti-O2 and anti-O12). The results obtained (Fig. 2B) were very similar to those obtained above in Fig. 2A, demonstrating the greater sensitivity of the O12 indicator over the O2 indicator.

**Figure 3:**
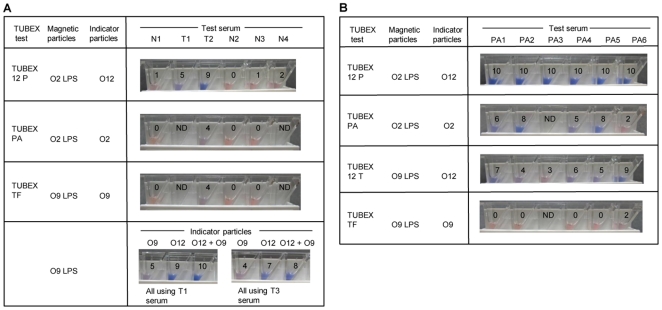
Pictoral results showing efficacy of various TUBEX tests in detecting clinical samples. Sera obtained from typhoid (A) or paratyphoid A (B) patients, or from healthy individuals (A), were examined as described in Figure 2. N1-N4, healthy individuals; T1, typhoid individual; T2, pool of 4 typhoid patients; T3, pool of 4 other typhoid patients; PA1-PA6, paratyphoid A patients; numericals in microwells denote TUBEX scores; ND, not done (insufficient specimen).

Third, we examined the performance of O12 indicator-based TUBEX tests in the examination of stored serum samples from 3 typhoid patients (Fig. 3A). In the 1^st^ specimen (T1), both TUBEX 12P (which primarily detects anti-O12 antibodies) and TUBEX TF (which primarily detects anti-O9 antibodies), were weakly positive (score 5), but TUBEX 12T was strongly positive (score 9) (see bottom of figure). Using both the O12 and O9 indicators together enhanced the reaction only marginally. The importance of anti-O12 antibodies to the reaction is also seen in specimen T2—higher readings in TUBEX 12P (score 9) than those of TUBEX PA or TUBEX TF (both, score 4). Similarly, in specimen T3, higher readings were obtained using the O12 indicator (score 7) instead of the O9 indicator (score 4). In all cases, the TUBEX tests were negative for sera obtained from healthy individuals.

Finally, O12 indicator-based TUBEX tests were used to examine stored sera from 6 paratyphoid A patients (Fig. 3B). Remarkably, TUBEX 12P detected all 6 sera at the highest intensity (score 10), whereas TUBEX PA detected these with significantly lower readings, especially in PA6 (score 2) and PA4 (score 5). This may be attributed to the additional detection of certain types of anti-O12 antibodies by TUBEX 12P (both tests can detect anti-O2 antibodies). Since TUBEX 12T detects the same anti-O12 antibodies as TUBEX 12P but not anti-O2 antibodies, the high scores seen in TUBEX 12P may be due to the complementation between TUBEX PA and TUBEX 12T. Thus, patient PA6 has a low TUBEX PA score of 2 but a high TUBEX 12T score of 9, while the converse is true of patients PA2 and PA5. Similar amounts of anti-O2 and anti-O12 antibodies appeared to be made in patients PA1 and PA4. TUBEX TF was negative for these cases, implying the absence of cross-reacting anti-O12 antibodies.

### TUBEX 12T detects typhoid better than TUBEX TF

Various TUBEX tests (see Fig. 1) were used to detect typhoid fever from 22 culture-confirmed typhoid patients who lived in Xinjiang Province in China. Paired sera were obtained from each patient with a time interval of 4 – 23 days between samples, and all sera were kept at −80°C for < 4 years before the present study.

As shown in Table S1, patients are designated as T31 – T73, while the 1^st^ and 2^nd^ serum samples from each patient are labeled ‘a’ and ‘b’, respectively. The samples are divided into 4 major groups (Group A – D) according to the IgM and IgG ELISA activities to *S*. Typhi LPS. Categorization was done after the TUBEX tests were performed, the latter independently of ELISA. Thus, Group A specimens have moderate (M) to high (H) levels of both IgM and IgG antibodies, while Group B specimens have M – H IgM, but low (L) to negligible IgG levels of antibodies. Group C specimens are virtually devoid of any antibodies to all 3 *Salmonella* antigens, while Group D specimens have no detectable IgM antibodies to *S*. Typhi LPS, but have high levels of IgG antibodies to all 3 antigens. Group A is further sub-divided into Group A1, in which specimens were obtained ≤ 11 days following onset of fever, and Group A2, in which the specimen was obtained after 17 days. Accordingly, Group A1 comprises only ‘a’ specimens, and Group A2 mostly ‘b’ specimens except for 2 ‘a’ specimens.

In Group A1 (10 specimens), TUBEX TF and TUBEX 12T detected 90% and 100% of the serum samples, respectively. However, the positive scores in TUBEX TF were modest, with only 30% of samples having scores ≥ 6, the highest score being 7. Better results were observed with TUBEX 12T (70% with scores ≥ 6, highest score being 8). Both paratyphoid A tests, TUBEX PA and TUBEX 12P, also detected the test samples with high efficiency (80% and 100%, respectively) and with positive scores slightly better than those of TUBEX TF. In all cases, TUBEX PA reactivity could be blocked completely by soluble *S*. Typhi LPS (see TUBEX bPA), which implicates the involvement of anti-O12 antibodies. In contrast, in TUBEX TF (see TUBEX bTF), the reaction was essentially unaffected by *S*. Paratyphi A LPS, suggesting that reactivity to be due largely to anti-O9 antibodies.

In Group A2 (17 specimens), both TUBEX TF and TUBEX 12T detected all cases except for one (94%). The odd specimen (T71b) came from a patient whose earlier (1^st^) specimen was also negative. Again, the positive scores from TUBEX 12T (76.5% with scores ≥ 6, highest score being 9) were superior to those of TUBEX TF (35.3% with scores ≥ 6, highest score being 7). TUBEX 12P also performed well (94% sensitive), but TUBEX PA was only 58.8% positive. Reactivity in TUBEX PA was completely abolished by soluble *S*. Typhi LPS in 17 cases, whereas that in TUBEX TF was virtually unaffected by soluble *S*. Paratyphi A LPS.

In Group B, both TUBEX TF and TUBEX 12T detected all 7 sera (100%). In contrast, none (0%) was detected by TUBEX PA, while TUBEX 12P was positive in 5 cases (71.4%). Higher scores were again obtained by TUBEX 12T over TUBEX TF. There are 2 pairs of sera in this group (from individuals T31 and T63), and in both cases, the 1^st^ specimen was obtained 8 days after fever onset, and the 2^nd^ specimen on day 22–23. Two other serum samples (from patients T57 and T65) were also early (day 8–10), while the remaining sample from patient T41 was obtained on day 24.

In Group C, which had virtually no ELISA antibodies to any of the antigens, the TUBEX results were similarly negative in virtually all 7 cases. There are 3 pairs of sera in this group, and in all cases, the 1^st^ specimen was obtained 7–9 days after onset of fever, and the 2^nd^ specimen on day 21–23. The remaining single serum was obtained on day 38, which came from patient T41 whose earlier serum sample, yielded strong reactivities in both ELISA and TUBEX (see Group B).

Group D comprises only 3 specimens, including the early (day 9) and late (day 22) serum from patient T47 — both were strongly positive in TUBEX 12T, TUBEX 12P and TUBEX PA, despite having no detectable ELISA IgM antibodies to *S*. Typhi LPS (but high IgG levels to all 3 antigens present). The remaining specimen was an early (day 7) sample with similar ELISA profile, and both this and a later (day 30) sample from the same patient (T71, see Group B) were negative in all TUBEX tests.

Taking the whole cohort of sera together (Groups A–D), the sensitivity of detection for IgM ELISA is 77.3% (34/44), compared to 75.0% (33/44), 45.5% (20/44), 81.8% (36/44) and 75.0% (33/44), for TUBEX TF, TUBEX PA, TUBEX 12T and TUBEX 12P, respectively. That is, TUBEX 12T is slightly better in sensitivity than either IgM ELISA or TUBEX TF. If only sera that are IgM ELISA-positive (i.e. Groups A and B) are considered, then the sensitivities of TUBEX 12T and TUBEX TF become 97.1% (33/34) and 94.1% (32/34), respectively.

**Figure 4:**
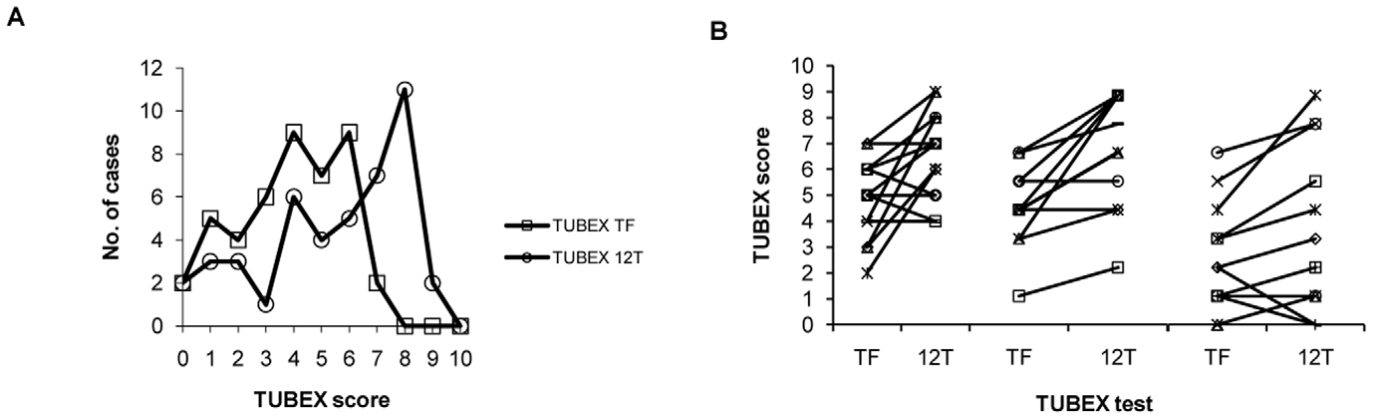
Comparison of the results scores between TUBEX TF and TUBEX 12T in typhoid patients. Based on the frequency of the various scores (0–10) (A), or on each individual patient (total 22 patients) (B).

Again, when considering the whole cohort of sera, TUBEX 12T is superior to TUBEX TF in terms of results score. Thus, only 25% (11/44) of the sera in TUBEX TF had scores ≥ 6, with the highest score being 7, compared to 56.8% (25/44) for TUBEX 12T, 52% (13/25) of these sera having scores of 8–9 (Fig. 4A). Increase in results scores from TUBEX TF to TUBEX 12T was observed in 59.0% (13/22) of individuals (Fig. 4B).

**Figure 5:**
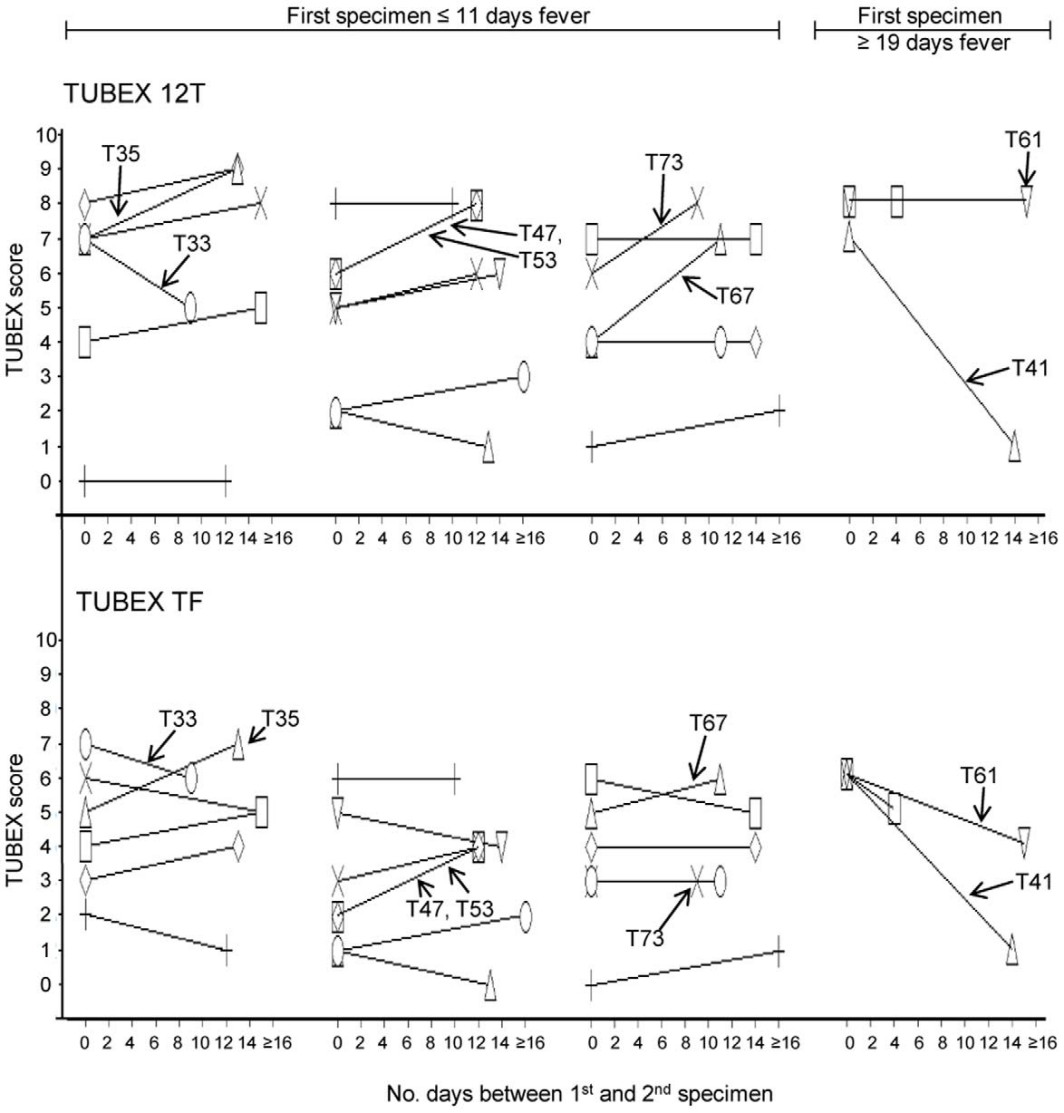
Change in antibody titer (TUBEX score) over time in individual typhoid patients. TUBEX 12T or TUBEX TF was used to examine the 1^st^ and 2^nd^ specimen from 22 patients. Cases divided between those whose 1^st^ specimens were obtained less than 11 days of fever, and those obtained after 19 days; indicated are ones showing significant rise or fall in antibody titer.

Fig. 5 summarizes the change in antibody titer between the 1^st^ and 2^nd^ specimen of all 22 typhoid patients, determined by TUBEX TF or TUBEX 12T. Results between the two tests are very similar. Remarkably, there was no significant change in TUBEX score in the majority (63.6% [14/22]) of patients (change defined as difference in TUBEX score ≥ 2 in either or both of the TUBEX tests). Increase in titer was observed in 5 individuals (26.3% [5/19]) whose 1^st^ specimen was obtained within 11 days of fever onset, and the 2^nd^ specimen after a further 8–12 days. Decrease in titer was observed in 3 patients, the 1^st^ specimen in 2 cases obtained after 19 days of fever, in one of whom (patient T41), a dramatic drop in TUBEX titer (from high to nil activity over 2 weeks) was observed.

**Figure 6:**
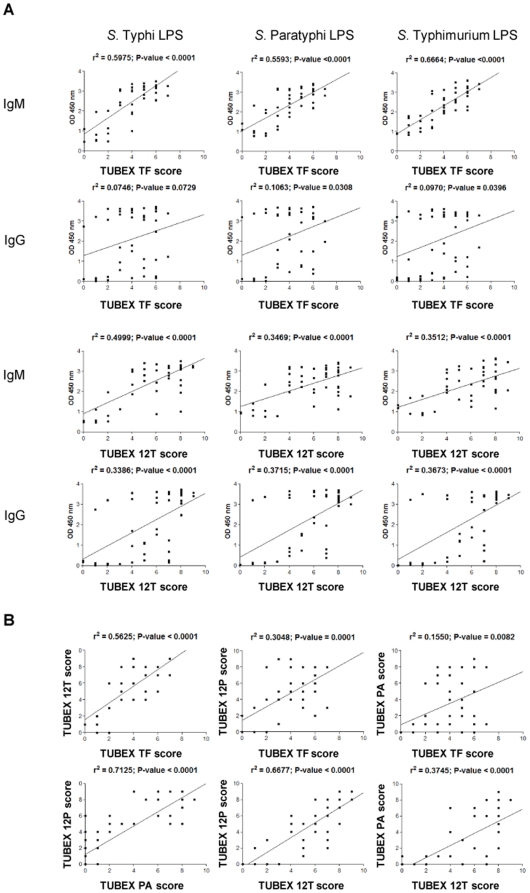
Regression analysis of various ELISA and TUBEX results obtained from typhoid patients. (Based on Table S1.)

Regression analysis was performed to examine the relationships between the various tests (Fig. 6). Thus, it appears that the TUBEX TF results are better correlated with those of IgM ELISA than IgG ELISA, regardless of which *Salmonella* LPS was used in the ELISA (e.g. *r^2^* = 0.598 *P*<0.001 for TUBEX TF vs IgM ELISA *S*. Typhi LPS; *r^2^* = 0.075 *P* = 0.073 for TUBEX TF vs IgG ELISA *S*. Typhi LPS). TUBEX 12T is similar to TUBEX TF in this regard, except that, in TUBEX 12T, best correlation was observed with IgM ELISA *S*. Typhi LPS (*r^2^* = 0.50 *P*<0.001), and there is also correlation with IgG ELISA (regardless of LPS type) (e.g. *r^2^* = 0.339 *P*<0.001 for TUBEX 12T vs IgG ELISA *S*. Typhi LPS).

It is also apparent that TUBEX TF is best correlated with TUBEX 12T (*r^2^* = 0.563 *P*<0.001) than with TUBEX 12P (*r^2^* = 0.305 *P* = 0.001) or TUBEX PA (*r^2^* = 0.155 *P* = 0.008). On the other hand, there is strong association between TUBEX 12P and TUBEX PA (*r^2^* = 0.713 *P*<0.001), and between TUBEX 12P and TUBEX 12T (*r^2^* = 0.668 *P*<0.001).

### TUBEX 12P detects paratyphoid A fever better than TUBEX PA

Similar to the above study with typhoid patients, herein the efficiencies of detection of paratyphoid A fever by various TUBEX and ELISA tests were compared using single serum samples from 36 culture-confirmed paratyphoid A patients who lived in Yunnan Province in China. Twenty-four of these sera were procured in 2006, and the other 12 sera in 2009; all sera were stored at −80°C.

Similar to the typhoid study, the whole cohort of sera is divided into 4 groups (A – D) based on the IgM and IgG ELISA activities to *S*. Paratyphoid A LPS (instead of *S*. Typhi LPS) (see Table S2). Again, this categorization was done independently of, and after the TUBEX tests. Group A is sub-divided into Group A1 and A2, in which the sera were procured in 2006 and 2009, respectively. Group D is similarly sub-divided into Group D1 and Group D2. A significant difference between this tabulation and that for the typhoid patients is the relatively large size of Group D (especially D1) individuals here. Another difference is that, many of the paratyphoid sera, especially those in 2006, were procured within the 1^st^ week of fever onset. In addition, unlike Group A typhoid patients who had high levels of both IgM and IgG ELISA activities to all 3 LPS antigens, 7 of 9 Group A1 paratyphoid patients had only low levels of IgM ELISA antibodies to *S*. Paratyphoid A (but most had high IgG levels), and no IgM ELISA antibodies to the other LPSs. Group A1 is, in fact, quite similar to Group D1.

Thus, in Group A1, TUBEX 12P detected 6 of the 9 individuals (66.7%), but TUBEX PA detected only 2 of them (22.2%). Both of the latter individuals had M – H levels of IgM ELISA antibodies to *S*. Paratyphoid A, and both yielded high results scores in TUBEX PA and TUBEX 12P.

All 6 Group A2 individuals, who had M – H levels of IgM ELISA antibodies to *S*. Paratyphoid A, were strongly detected by TUBEX 12P (scores 6 – 9). Most were also detected by TUBEX PA (83.3%, scores 3–10), but only 50% (scores 4 – 7) by TUBEX TF.

Group B comprises a single individual with low IgM ELISA antibodies who was negative in both TUBEX 12P and TUBEX PA. All 4 Group C patients had no ELISA antibodies and were negative in TUBEX PA (1 individual, marginally positive) and TUBEX 12P.

Group D1 sera generally lacked IgM ELISA antibodies to the 3 LPS antigens but contained abundant levels of IgG antibodies. In Group D1, TUBEX 12P detected 4 of 12 sera (33.3%), and TUBEX PA only 2 (16.7%). In Group D2, slightly better results were obtained here: 50% (2/4) for TUBEX 12P, and 25% (1/4) for TUBEX PA.

Considering the whole cohort of sera, the IgM ELISA (*S*. Paratyphi LPS) (15/36 or 41.7%) is slightly less sensitive than TUBEX 12P (52.8%). Considering the 2009 specimens only, the corresponding statistics become 50.0% (6/12) and 66.6% (8/12), respectively. Similarly, for 2006, the statistics are 41.7% (10/24) and 45.8% (11/24), respectively.

### A common TUBEX test for typhoid/paratyphoid A fever and a typhoid–specific TUBEX TF test

Based on the above findings, we investigated the possibility of combining TUBEX 12T with TUBEX 12P to establish an efficient test to detect both typhoid and paratyphoid A fever simultaneously. Only a preliminary study was conducted due to shortage of test material, using available sera from a few typhoid and paratyphoid A patients only. Indeed, we found that TUBEX 12TP, which is comprised of equal proportions of TUBEX 12T and TUBEX 12P: (a) detected purified anti-O12 mAb (mAb P4E8) as efficiently (32 µg/ml) as TUBEX 12T and TUBEX 12P, and (b) detected 9 of 10 typhoid and all 3 paratyphoid sera examined (Table S3). The results scores were similar to, or marginally lower than, the corresponding results in TUBEX 12T and TUBEX 12P for the typhoid and paratyphoid patients, respectively, but were generally better than those in TUBEX TF and TUBEX PA for the respective patients. However, in one particular paratyphoid patient (P12), the TUBEX 12TP determination (score 4) was significantly lower than that of TUBEX 12P (score 8). To redress this problem, we modified the combined test using twice as much TUBEX 12P as TUBEX 12T, and in this new test (TUBEX 12TPP) with this particular serum, an improvement was indeed noted (score 6). Importantly, in TUBEX 12TPP, the results scores for the typhoid sera remained essentially the same as those in TUBEX 12TP.

We further investigated the possibility of modifying TUBEX TF to make it truly typhoid–specific, suitable as a differential test used in conjunction with TUBEX 12TPP. Such a modification (bTF) was found possible, by incorporating soluble *S*. Paratyphi A LPS in the test as an adsorbent to remove anti-O12 antibodies (Table S1). However, as mentioned, the problem with the existing TUBEX TF test is that the results scores are generally weaker than those of TUBEX 12T, and consequently, a weak typhoid case could be misinterpreted as negative. To overcome this problem, we investigated the possibility of enhancing the reaction intensity in TUBEX TF by using twice as much serum sample (with half the vol of indicator particles, of a higher concentration). Experiments using purified anti-O9 mAb (3h1) indicate that this modification was feasible (Table S4A). Confirmation was found using 3 typhoid serum samples, in which TUBEX TF scores of 5-6 were obtained instead of 2-4 (Table S4B). Moreover, in the presence of *S.* Paratyphi A LPS (blocker), the results of the modified method (bTF) for these sera remained distinctly positive (score 4 in all cases), while those for 6 individual paratyphoid sera used as control were negative (score 0 – 2).

In the study, we also examined the specificity of the TUBEX tests using sera obtained from 18-20 healthy individuals and 6-10 patients with non-*Salmonella* febrile illness (Table S5). None of the subjects were positive in TUBEX PA and TUBEX 12P, but 3-4 subjects were marginally positive (score 3–4) in TUBEX TF or TUBEX 12T. All 3 positive cases in TUBEX TF also had low IgM ELISA activity to *S*. Typhi, 2 of whom were also positive in TUBEX 12T. Only 4 specimens were available for subsequent testing by TUBEX 12TP and TUBEX 12TPP—all were negative (score 0) in these tests.

## Discussion

We examined the assay characteristics of TUBEX TF and several prototypic TUBEX tests (TUBEX PA, TUBEX 12T, TUBEX 12P, TUBEX 12TP [including the variant, TUBEX 12TPP]) in the detection of typhoid and paratyphoid A fever. These tests were designed to detect anti-O9 (TUBEX TF), anti-O2 (TUBEX PA) or anti-O12 (TUBEX 12T, TUBEX 12P, TUBEX 12TP) antibodies by direct inhibition of binding of the reagent mAb to its target (see Fig. 1). As revealed both here and in a previous study [18], these tests not only detected the intended antibody, but also indirectly, by virtue of steric hindrance, antibodies that bind to a neighboring epitope. That is, anti-O12 antibodies from patients are cross-detected in TUBEX TF and TUBEX PA, while anti-O9 antibodies are similarly detected in TUBEX 12T and TUBEX 12TP, and anti-O2 antibodies in TUBEX 12P and TUBEX 12TP. Thus, both the anti-O9 (3h1) and anti-O2 (P1D10) mAbs clearly inhibited binding of the anti-O12 indicator particle in TUBEX 12T, TUBEX 12P and TUBEX 12TP. However, unexpectedly, the anti-O12 mAb (P4E8) was not inhibitory for the anti-O9 (TUBEX TF) or anti-O2 (TUBEX PA) indicator. Since, on the other hand, studies using sera from both typhoid and paratyphoid A patients clearly showed anti-O12 antibodies to be present that were responsible for the cross-detection in TUBEX TF and TUBEX PA, we reason that patients can produce more than one type of anti-O12 antibodies. This may not be surprising since the O12 antigen is relatively large, being comprised of a trisaccharide. This may also explain the apparent abundance of anti-O12 antibodies in typhoid and paratyphoid patients. A simplistic view is, there are at least 3 types of anti-O12 antibodies, one to each of the 3 sugars, and all 3 types can inhibit binding of the indicator anti-O12 mAb to its antigen (Fig. 1). Only one type (anti-O12a), however, is capable of blocking the anti-O9 or anti-O2 indicator. The high clinical sensitivity of O12-based TUBEX tests may thus be attributed to the strategic location of the antigenic epitope (O12b) in the LPS to which mAb P4E8 binds.

Accordingly, in both the typhoid and paratyphoid patients, the anti-O12 detection tests (TUBEX 12T and TUBEX12P) detected more cases than the anti-O9 (TUBEX TF) or anti-O2 (TUBEX PA) test. More impressively, these tests also yielded significantly higher results scores in the majority of patients than the corresponding tests. Thus, in the case of typhoid patients, whereas only about a quarter of the subjects obtained the highest TUBEX TF scores of 6–7, roughly the same proportion obtained scores of 8–9 in TUBEX 12T. Since both tests are similar in sensitivity in detecting mAb P4E8, and both detect a common anti-O9 antibody population, very likely, the superiority of TUBEX 12T is due to the additional detection of anti-O12b and anti-O12c antibodies. Regression analysis supports this, and in addition, it seems that IgG antibodies are also involved. Thus, enhanced TUBEX 12T scores are best seen in ELISA Group A2 (late-stage) individuals who have high ELISA levels of both IgM and IgG classes, whereas in Group B individuals who have IgM but no IgG antibodies, there is little difference between the TUBEX 12T and TUBEX TF scores.

We demonstrated that it is possible to combine TUBEX 12T with TUBEX 12P to produce a combined test (TUBEX 12TP) for typhoid and paratyphoid fever. Such a test is desirable since both diseases require similar clinical attention, and this can save resources, time, and clinical material. The most important requirement is that such a test can distinguish these *Salmonella* infections from other non-*Salmonella* febrile diseases such as dengue fever and malaria. Since O12 is unique to *Salmonella*, and based on the limited observation with control sera in the present study as well as from previous TUBEX TF findings [17], TUBEX 12TP seems to have the desired specificity. By mixing different proportions of TUBEX 12T and TUBEX 12P together to achieve the desired O9:O2 antigenic ratio (O12 remaining constant), we found that TUBEX 12TPP may be better suited for paratyphoid A detection due to the apparently higher amounts of anti-O2 antibodies produced than anti-O12 antibodies in some patients. While TUBEX 12TP (or 12TPP) is intended to be used as a screening test for both typhoid and paratyphoid A, it actually casts a wider net and captures paratyphoid B as well, including infections caused by other *Salmonella* members belonging to serogroup A, B and D, that manage to invade the bloodstream and stimulate a systemic antibody response. However, most of the time and in most places, it will be typhoid and paratyphoid A that will be responsible for the reactivity. If, for epidemiological reasons there is need to distinguish between these two diseases, we showed that this can be done by re-testing the specimen in TUBEX TF in the presence of a blocker (*S*. Paratyphi A LPS) to remove the anti-O12 antibodies. Sera that contain anti-O9 antibodies i.e. from typhoid patients, will be positive in the differential test.

An extremely important inclusion in the study is the use of ELISA to characterize the sera independently and objectively. Though rather cumbersome and time-consuming for routine use, this highly sensitive test quantifies the amount of IgM or IgG antibodies to *S*. Typhi (O9^+^ O12^+^) LPS, *S*. Paratyphi A (O2^+^ O12^+^) LPS, or *S*. Typhimurium (O4^+^ O12^+^) LPS. Thus, the relevant ELISA results found in the present study correlated very well with those of the TUBEX tests, corroborating the fact that TUBEX reactions are truly antibody-mediated. Regression analysis suggests that IgM antibodies, rather than IgG antibodies, are responsible for the TUBEX inhibitions. This agrees well with a previous experimental study [19] and a clinical study [12]; however, the latter also indirectly implied that IgG antibodies, when present in great abundance, can facilitate the inhibitory activity of the IgM antibodies. In the present study, a similar supporting role of IgG antibodies may explain the enhanced TUBEX 12T scores seen in some patients, including patients (e.g. T47) who seemed to have little or no IgM ELISA antibodies.

The IgM ELISA results correlated well with those of TUBEX TF or TUBEX 12T regardless of LPS or TUBEX type, suggesting that anti-O12 antibodies are predominantly detected by the TUBEX tests. However, only a subset of these antibodies (anti-O12a) is detected by TUBEX TF, and these antibodies are also responsible for the cross-detection of typhoid patients by TUBEX PA and TUBEX 12P. Thus, these results strongly argues against any claim that ELISA or lateral-flow tests based on *S*. Typhi or *S*. Paratyphi A LPS, or the O-antigen Widal tests, can discriminate between typhoid and paratyphoid A fever.

Categorization of the study cohort into the 4 ELISA groups allows a greater insight into disease pathogenesis and a more precise assessment of TUBEX performance. Thus, it appears that Group B represents a very early stage in typhoid, since IgM antibodies, but no IgG antibodies, were made to *S*. Typhi. Accordingly, the relevant specimens were procured early in the infection (8–10 days), and secondly, IgG antibodies were indeed found in later specimens (Group A2) obtained from 2 patients (T57 and T65). However, in 2 other patients (T31 and T63), IgM antibodies only (without IgG) were also found in the 2^nd^ specimen obtained 2 weeks after the first. This suggests an absence of class-switching. This is also apparent in patient T41, but interestingly in this case, the 2^nd^ specimen (see Group C) obtained 2 weeks later showed a total absence of antibody activity. Thus, altogether, the findings suggest that it may not be uncommon (3/22 or 13.6%) to find individuals who mount an antibody response to *Salmonella* LPS antigens that fails to undergo class-switching (like a true thymus-independent response), and seemingly, the response then disappears completely. To our knowledge, this intriguing observation has not been reported previously for any disease. From a diagnostic viewpoint, these early specimens are very efficiently detected by both TUBEX TF and TUBEX 12T, implying that IgM anti-O9 antibodies are made abundantly early in infection. In stark contrast, no anti-O12a antibodies seem to be made at this stage since TUBEX PA was negative for all samples examined. On the other hand, IgM anti-O12b or anti-O12c antibodies are produced since TUBEX 12P was positive for some of the cases, and IgM ELISA was positive for all LPS types.

Group A1 probably also represents an early disease stage, but here, the antibody response has undergone normal class-switching. Thus, both IgM and IgG anti-O9 antibodies were produced – hence, the high efficiency of detection by both TUBEX TF and TUBEX 12T. It is possible that patients in this group had a previous exposure to *S*. Typhi organisms, and the rapid IgG production is due to an anamnestic response. Also produced in these patients are IgM anti-O12a and other anti-O12 antibodies, which could account for the high detection rates of TUBEX PA and TUBEX 12P.

Group A2 probably represents a disease stage subsequent to that of Group A1 – convalescence. Accordingly, TUBEX TF, TUBEX 12T and TUBEX 12P all performed very well here. Surprisingly, however, TUBEX PA was only moderately sensitive. A possible explanation is, either the anti-O12a antibody response is terminated in some individuals, or IgG anti-O12a antibodies are now made that do not inhibit in TUBEX PA.

Group C individuals (3 patients with 3 serum pairs) produced little or no ELISA or TUBEX antibodies to any of the *Salmonella* antigens. The most likely explanation is that these individuals are naturally (genetically) non-responders; alternatively, the offending organisms in these people were eliminated by early antibiotic treatment before they could stimulate the immune response.

It is more difficult to explain the ELISA profile (nil-IgM, high IgG) in Group D. One possibility is that the IgM antibodies, which are more fragile than IgG antibodies [20], are denatured due to storage of the specimen. Alternatively, in the case where both the 1^st^ and 2^nd^ specimens share a similar ELISA profile, this may be related to antibody production.

The foregoing suggests the possibility that there is a temporal pattern of antibody production to the various LPS antigens. Thus, the anti-O9 response appears to be initiated very early during infection, and is sustained for a long time. Others, such as the anti-O12a response, seem to develop later and are prone to change or early termination. Several examples can be found in the study cohort which show either a rise or fall in antibody titers in TUBEX TF or TUBEX 12T over a 4–16 day period (Fig. 5). As expected, antibody increase was observed in cases in which the 1^st^ specimens were obtained early in infection, whereas antibody decrease was usually associated with late-phase 1^st^ specimens. However, a surprising finding is that, the majority (64%) of patients showed no significant change in TUBEX or ELISA titers over time (Fig. 5). This is an important revelation, as it challenges the belief that use of paired sera in the Widal test is superior to using single serum samples.

Similar analysis can be made of the paratyphoid ELISA groups. The sera here were obtained from a different region from the typhoid sera, and unusually, most were obtained in the 1^st^ week of disease. One striking difference between these sera and the typhoid sera is the relatively large group of paratyphoid patients in Group D who generally made IgG antibodies, but no IgM antibodies, to the 3 *Salmonella* antigens. Furthermore, unlike Group A typhoid patients who produced vast amounts of both IgM and IgG antibodies to all 3 antigens, Group A1 paratyphoid patients produced only small amounts of IgM antibodies (but high IgG) - very similar, in fact, to Group D individuals.

Thus, one possibility is that many of the Group A1 and Group D paratyphoid sera had deteriorated, which affected the more fragile IgM antibodies but not the IgG antibodies. Alternatively, these are early-stage sera obtained from hosts in whom IgM production had not begun; the unexpected presence of IgG antibodies could perhaps be explained by previous vaccination against *S*. Typhi.

In favor of deterioration is the observation that paratyphoid sera obtained more recently in 2009 (Group A2 and D2) generally performed better in the TUBEX tests than those obtained in 2006 (Groups A1 and D1) (see below). Indeed, sera procured even more recently (2010), from a different geographical region, were found to be efficiently detected by TUBEX 12TPP (our unpublished observations). In addition, contrary to expectation, several serum samples obtained very early in disease in 2006 (e.g. P01, P12, P19) exhibited strong TUBEX 12P scores, while several others obtained very late in disease (e.g. P07, P11, P20), were negative in the test.

Furthermore, the overall sensitivities of detection of the whole cohort of paratyphoid patients by TUBEX 12P and TUBEX PA (52.8% and 28.6%, respectively) are significantly lower than those for typhoid patients by TUBEX 12T and TUBEX TF (81.8% and 75.0%, respectively). This is puzzling since TUBEX 12T and TUBEX 12P, for instance, have very similar assay sensitivities. Inclusion of an independent, objective test in the study was instructive. Thus, similar differences were also found in the IgM ELISA between the typhoid (77.3%) and paratyphoid (44.4%) sera, including the high concordance between the TUBEX and ELISA results for individual cases, suggesting that the problem lies in the specimen and not the test. A surprising finding here is that the overall ELISA results are, in fact, marginally poorer than the corresponding TUBEX results. The exceedingly low detection rate by TUBEX PA of the paratyphoid patients stands in stark contrast to previous findings with the test [18], again implicating specimen as the possible cause of the difference.

If only sera that are IgM ELISA-positive are considered, then the sensitivities of TUBEX 12T and TUBEX 12P increase to 97.1% and 75.0% for the typhoid and paratyphoid patients, respectively. Moreover, if the IgM-positive paratyphoid sera are subdivided into those obtained in 2009 (Group A2) or 2006 (Group A1), it is evident that TUBEX 12P can be very sensitive if given the appropriate specimen (100%, 2009 specimens).

Another factor often taken for granted which can also adversely affect the true worth of antibody tests is the use of culture as the gold standard in diagnosis. Based on this yardstick, antibody tests cannot be any more sensitive than culture; in fact, they are more likely to be inferior because culture-positive cases are often inadvertently selected from the early phase of the disease when the organisms circulate freely in the blood while antibody production may have only just begun. The problem here is, culture itself is generally known to be only about 60% or less sensitive; hence, there exists a potentially large group of true typhoid patients who are culture-negative but who could be antibody-positive — patients in late-phase disease, for example. How big this group is will determine the real sensitivity of the antibody tests — and that of culture. It is highly possible that antibody tests can indeed be more sensitive than culture and a better indicator of infection. We do not have such a group in the present study but previously [11], we have identified several individuals of the said description. We have also seen similar cases that are apparently healthy (non-febrile) and in whom the TUBEX TF reactions were confirmed to be real by O9 LPS absorption. These could be individuals who have a subclinical or asymptomatic *S*.Typhi infection, in whom disease is modulated by the same protective antibodies that also serve as diagnostic markers (e.g. anti-O9), or people who are chronic typhoid carriers.

Similarly, if the group of culture-negative, antibody-positive febrile patients is considered as control-negative in a study without further qualification, estimation of assay specificity will be adversely affected because such cases will be regarded as false-positives. One way to avoid this uncertainty, as demonstrated elegantly by Ley *et al* [17], is to use as control typhoid-negative, a group that is not only negative for *S.*Typhi culture, but also culture-positive for some other organism presumed to be the cause of the febrile illness. Thus, based on such a group comprising 106 randomly-selected individuals, they found a specificity of 89% using TUBEX TF. Organisms grown were varied, but *Salmonella* organisms other than *S.*Typhi were obtained from 49 individuals. Instructively, when this sub-group was omitted from the calculation, the specificity rose to 97%. TUBEX TF was positive in 18% of these cases — we suspect the organisms found in these individuals might be *S*.Enteriditis or other *Salmonella* members that possess the O12 or O9 antigen, or both.

## Materials and Methods

### Study populations

Twenty-two culture-confirmed typhoid patients who lived in Xinjiang Province in China, and 36 culture-confirmed paratyphoid A patients from Yunnan Province in China, were used. All patients presented with fever for at least 3 days in the 1^st^ week of illness at the time of the hospital or clinic visit in 2006 or 2009. Patients provided written informed consent for participation in the study and blood from each patient was obtained by venipuncture according to guidelines approved by the Institutional Review Board in Xinjiang or Yunnan, China. The typhoid patients recruited in 2006, aged 7 to 19 years (median, 14.5 years), had fever for an average of 8.5 days (range, 7–35 days). The 24 paratyphoid A patients recruited in 2006, 16 to 59 years old (median, 31.5 years), had fever for 4.0 days (range, 2–44 days); while 12 other paratyphoid A patients from 2009, aged 15 to 64 years (median, 22.5 years), had fever for 7.5 days (range, 5–12 days). Twenty healthy healthcare workers from Beijing and 10 non-Salmonella febrile patients from Hebei, China, were used as control subjects.

Sera used in the preliminary studies were the same specimens obtained from typhoid and paratyphoid A patients described previously [18].

### Blood culture

Blood (4 – 8 mL) from adult subjects (≥12 years) was inoculated into BD Bactec Plus Aerobic/F culture broth (Becton Dickinson, Sparks, MD), that (2–4 mL) from children into BD Bactec Peds Plus/F medium (BD). The broth was incubated aerobically at 37°C; when growth was apparent, or after 7 days, subculture was made onto blood and MacConkey agar. *Salmonella*-suspect colonies were identified by standard biochemical testing and, where appropriate, serotyped by slide agglutination using *Salmonella* O and H group-specific antisera obtained from the Chengdu Institute of Biological Products, China.

### TUBEX tests

- Antibody-coupled indicator particles: Anti-O2 and anti-O12 mAb produced previously [18], was coupled to blue latex particles (1.0 µm; Merck) by passive adsorption as described previously [21]. Briefly, equal volumes of antibody (1 mg/mL) and latex particles (1% w/v suspension) were mixed in 0.1M glycine buffer (pH 8.2) containing 0.9% NaCl (GBS buffer) and incubated on a roller at 4°C for 16 hr. The particles were blocked with 2% BSA in GBS buffer and then washed twice with distilled water containing 0.3% Tween 20, and once with GBS buffer containing 1% BSA. Finally, the particles were resuspended in 1% BSA-GBS buffer.
- LPS antigen-coupled magnetic particles: O9 LPS was obtained from Sigma Chemical, St. Louis, MO, and O2 LPS was prepared by phenol extraction as described previously [18]. LPS (0.6–1.0 mg/mL) was coupled to magnetic latex particles (0.8 µm; Merck) by passive adsorption. The coupling concentration of O9 LPS and O2 LPS was 1 mg/mL and 0.63 mg/ml, respectively [21].
  TUBEX TF was obtained from IDL Biotech, Sollentuna, Sweden; the Blue and Brown reagents from this kit were also used in the prototypic TUBEX tests described in the study (TUBEX PA, TUBEX 12T, TUBEX 12P, TUBEX 12TP and TUBEX 12TPP).
- Procedure: All tests were performed according to the manufacturer's instructions for TUBEX TF, except that a smaller vol of reagents or specimen was used. Briefly, 25 µL of Brown reagent (antigen-coupled magnetic particles) were placed in a well of a specially-designed reaction vessel (made up of 6 small, identical V-shaped wells). The test serum (25 µL) was then added and mixed with the Brown reagent using a pipette. The mixture was stood for 2 min. Blue reagent (antibody-coupled indicator particles; 50 µL) was then added, and the whole set of reaction wells was sealed with adhesive tape and shaken rapidly for 2 min in an automatic portable shaker (TUMIX, IDL Biotech). The set of reaction wells was then stood for about 2 min on the magnet stand supplied, and the resulting color of the supernatant read by eye and scored against the color chart provided. The results were graded from 0 (red, most negative) to 10 (blue, most positive), with scores ≤ 2 considered as negative in the study.

The differential TUBEX tests (TUBEX bTF, TUBEX bPA and TUBEX bTFX) were performed as described previously [18]. Briefly, 5 µL (TUBEX bTF and TUBEX bPA) or 10 µL (TUBEX bTFX) LPS blocker (10 mg/mL *S.* Typhi LPS or *S*. Paratyphi A LPS in GBS buffer) or GBS buffer (control) were added to the reaction well containing the Brown reagent, and the rest of the procedure then followed as described above.

### Anti-LPS ELISA

The direct ELISA tests used were described previously [18]. Briefly, Immunolon-2 microplates (Thermo, Milford, MA) coated with *S*. Typhi LPS (1 µg/mL; Sigma Chemical, St. Louis, MO), *S*. Paratyphi A LPS (1 µg/mL; own production), or *S*. Typhimurium LPS (1 µg/mL; Calbiochem, La Jolla, CA) were incubated with patient's serum (1∶100 dilution) for 30 min at room temperature. The plates were washed and incubated with horseradish peroxidase-labeled mouse anti-human IgG or goat anti-human IgM (µ) (Invitrogen, Camanillo, CA) for 30 min at RT. After washing, substrate (3,3′, 5,5′ – tetramethylbenzidine) was added and incubated for 10 min at RT. Results were read at 450 nm in a TECAN Sunrise reader. Positive results were scored as low (“L”), medium (“M”), or high (“H”) with respect to the ELISA levels found in healthy subjects: mean + 1 SD ≤ L < mean + 2 SD; mean + 2 SD ≤ M < mean + 4 SD; H ≥ mean + 4 SD.

### Statistics

Regression analysis was performed using Prism 3 (Graph-Pad Software); *P*<0.05 was considered significant.

## Supporting Information

Table S1 **Antibody activity of sera from culture-confirmed typhoid patients determined by various ELISA and TUBEX tests.**
(PDF)

Table S2 **Antibody activity of sera from culture-confirmed paratyphoid A patients determined by various ELISA and TUBEX tests.**
(PDF)

Table S3 **Comparison of assay performance among various TUBEX tests in the detection of (A) purified mAb P4E8, and (B) various typhoid and paratyphoid sera.**
(PDF)

Table S4 **Effect of using double-volume specimen in TUBEX TF (TUBEX TFX) in the detection of (A) purified mAb 3h1, and (B) various typhoid and paratyphoid sera.**
(PDF)

Table S5 **Antibody activity of sera from healthy controls or non-** ***Salmonella*** **febrile patients determined by various ELISA and TUBEX tests.**
(PDF)
