# Supplementary material for: Combined Rapid (TUBEX) Test for Typhoid-Paratyphoid A Fever Based on Strong Anti-O12 Response: Design and Critical Assessment of Sensitivity
Source: PLoS One. 2011 Sep 15;6(9):e24743. doi: 10.1371/journal.pone.0024743 (PMC3174194; doi:10.1371/journal.pone.0024743)
Supplement: Table S1 — Antibody activity of sera from culture-confirmed typhoid patients determined by various ELISA and TUBEX tests. (PDF) [file pone.0024743.s001.pdf]

Table S1 Antibody activity of sera from culture-confirmed typhoid patients determined by various ELISA and TUBEX tests.

		ELISA IgM			ELISA IgG			TUBEX					
Specimen no.	Days of fever at sampling	**T-LPS**	P-LPS	M-LPS	**T-LPS**	P-LPS	M-LPS	**TF**	bTF	**PA**	bPA	**12T**	12P
Group A1 (≤ 11 days after fever onset)													
T33a	10	M	H	H	L	H	M	7	6	1	1	7	3
T35a	9	H	H	H	H	H	H	5	ND	6	0	7	6
T39a	8	H	M	L	H	H	H	3	ND	6	0	8	9
T45a	8	H	M	M	H	H	H	6	ND	8	0	7	7
T53a	11	M	M	L	H	H	H	2	ND	2	0	6	4
T55a	10	H	H	H	H	H	H	6	ND	9	0	8	8
T59a	10	H	H	H	L	M	M	5	ND	3	0	5	5
T67a	8	H	M	L	H	H	H	5	ND	4	0	4	5
T69a	8	M	M	M	H	H	H	4	3	7	0	4	6
T73a	8	M	L	-	H	H	H	3	ND	7	0	6	5
Group A1 sensitivity								9/10<br>**90.0%**		8/10<br>**80.0%**		10/10<br>**100%**	10/10<br>**100%**
Group A2 (≥ 17 days after fever onset)													
T33b	19	M	M	M	L	M	M	6	ND	0	0	5	2
T35b	22	H	H	H	H	H	H	7	ND	8	0	9	8
T37a	24	H	H	H	H	H	H	6	ND	2	0	8	5
T37b	28	H	H	M	H	H	H	5	5	2	0	8	5
T39b	21	H	M	L	H	H	H	4	5	8	0	9	9
T45b	23	H	H	M	H	H	H	5	ND	8	0	8	8
T53b	23	H	H	M	H	H	H	4	4	4	0	8	9
T55b	20	H	H	H	H	H	H	6	ND	8	0	8	7
T57b	20	H	H	M	M	H	H	4	4	2	0	6	4
T59b	24	H	H	M	M	H	M	4	4	2	0	6	6
T61a	35	M	M	L	H	H	H	6	6	5	0	8	8
T61b	50	M	M	L	H	H	H	4	ND	5	0	8	8
T65b	21	H	H	M	M	L	-	3	ND	1	0	4	4
T67b	19	H	H	M	H	H	H	6	ND	8	0	7	8
T69b	22	M	M	L	H	H	H	4	2	7	0	4	5
T71b	30	M	M	L	H	H	H	1	ND	1	0	2	3
T73b	17	M	-	-	H	H	H	3	ND	8	0	8	5
Group A2 sensitivity								16/17<br>**94.1%**		10/17<br>**58.8%**		16/17<br>**94.1%**	16/17<br>**94.1%**

Combined Group A sensitivity								25/27 **92.6%**		18/27 **66.7%**		26/27 **96.3%**	26/27 **96.3%**
Group B													
T31a	8	H	H	M	-	-	-	4	ND	1	1	4	4
T31b	23	M	H	M	-	L	-	5	ND	0	0	5	3
T41a	24	H	H	H	-	-	-	6	ND	0	0	7	6
T57a	8	H	M	L	-	M	M	3	ND	0	0	5	1
T63a	8	H	H	M	-	-	L	6	ND	1	0	7	2
T63b	22	M	H	M	-	L	L	5	ND	0	0	7	4
T65a	10	H	H	M	L	-	-	3	ND	1	0	4	4
Group B sensitivity								7/7 **100%**		0/7 **0%**		7/7 **100%**	5/7 **71.4%**
Group C													
T41b	38	-	-	L	-	-	-	1	ND	0	0	1	0
T43a	9	-	-	-	-	-	-	2	ND	1	0	0	0
T43b	21	-	-	-	-	-	-	1	ND	0	0	0	0
T49a	7	-	-	-	-	-	-	1	ND	1	0	2	0
T49b	23	-	-	-	-	-	-	2	ND	1	0	3	0
T51a	8	-	-	-	-	-	-	1	ND	0	0	2	0
T51b	21	-	-	-	-	-	-	0	ND	0	0	1	0
Group C sensitivity								0/7 **0%**		0/7 **0%**		1/7 **14.3%**	0/7 **0%**
Group D													
T47a	9	-	M	-	H	H	H	2	ND	6	0	6	8
T47b	22	-	M	L	H	H	H	4	2	7	0	8	9
T71a	7	-	L	-	H	H	H	0	ND	0	0	1	2
Group D sensitivity								1/3 **33.3%**		2/3 **66.7%**		2/3 **66.7%**	2/3 **66.7%**
Group (A + B) sensitivity								32/34 **94.1%**		18/34 **52.9%**		33/34 **97.1%**	31/34 **91.2%**
Group (A + B + C) sensitivity								32/41 **78.0%**		18/41 **43.9%**		34/41 **82.9%**	31/41 **75.6%**
Group (A + B + C + D) sensitivity								33/44 **75.0%**		20/44 **45.5%**		36/44 **81.8%**	33/44 **75.0%**

Results expressed as ELISA grades (-, negative; L, low; M, moderate; H, high antibody levels) or TUBEX scores (0-to-10, most negative-to-most positive). Specimen ‘a’ and (shaded) ‘b’ denote 1st and 2nd specimen, respectively, from same individual; T-LPS, P-LPS and M-LPS denote LPS from *S*. Typhi, *S*. Paratyphi A and *S*. Typhimurium, respectively; bTF is TUBEX TF with added soluble *S*. Paratyphi A LPS (blocker); bPA is TUBEX PA with added soluble *S*. Typhi LPS (blocker); ND = not done.
