# Supplementary material for: Combined Rapid (TUBEX) Test for Typhoid-Paratyphoid A Fever Based on Strong Anti-O12 Response: Design and Critical Assessment of Sensitivity
Source: PLoS One. 2011 Sep 15;6(9):e24743. doi: 10.1371/journal.pone.0024743 (PMC3174194; doi:10.1371/journal.pone.0024743)
Supplement: Table S2 — Antibody activity of sera from culture-confirmed paratyphoid A patients determined by various ELISA and TUBEX tests. (PDF) [file pone.0024743.s002.pdf]

Table S2 Antibody activity of sera from culture-confirmed paratyphoid A patients determined by various ELISA and TUBEX tests.

	ELISA IgM			ELISA IgG			TUBEX		
Specimen no.	T-LPS	**P-LPS**	M-LPS	T-LPS	**P-LPS**	M-LPS	**TF**	PA	**12P**
Group A1 (obtained in 2006)									
P01	M	M	L	M	H	H	ND	6	8
P02	-	L	-	L	H	M	ND	0	4
P06	-	L	-	L	H	M	ND	0	3
P08	-	L	-	L	H	-	ND	1	5
P09	-	L	M	M	M	M	ND	0	0
P12	-	L	-	M	H	H	ND	1	8
P16	-	L	-	H	H	M	ND	0	1
P19	H	H	H	H	H	H	ND	6	9
P24	-	L	-	-	L	-	ND	0	0
Group A1 sensitivity								2/9<br>**22.2%**	6/9<br>**66.7%**
Group A2 (obtained in 2009)									
P25	M	H	L	H	H	H	6	4	6
P27	M	H	H	H	H	H	4	8	9
P28	-	M	-	L	H	H	2	0	6
P30	M	H	M	H	H	H	7	10	9
P33	-	M	-	H	H	H	0	6	8
P36	L	H	-	H	H	H	0	3	6
Group A2 sensitivity							3/6<br>**50.0%**	5/6<br>**83.3%**	6/6<br>**100%**
Combined Group A sensitivity							3/6<br>**50.0%**	7/15<br>**46.7%**	12/15<br>**80.0%**
Group B									
P04	-	L	L	-	-	M	ND	0	0
Group B sensitivity								0/1<br>**0%**	0/1<br>**0%**
Group C									
P05	-	-	-	-	-	-	ND	0	0
P10	-	-	-	-	-	-	ND	1	4
P31	-	-	-	-	-	-	0	0	0
P35	-	-	-	-	-	-	0	0	0

Group C sensitivity							0/2<br>**0%**	0/4<br>**0%**	1/4<br>**25.0%**
Group D1 (2006)									
P03	-	-	-	M	H	H	ND	2	6
P07	-	-	-	-	M	H	ND	0	2
P11	-	-	-	M	H	H	ND	0	3
P13	-	-	-	-	H	H	ND	0	0
P14	-	-	-	L	H	H	ND	7	7
P15	-	-	L	L	L	-	ND	0	0
P17	-	-	-	H	H	H	ND	0	1
P20	-	-	-	-	M	M	ND	0	0
P21	-	-	-	M	M	H	ND	0	0
P22	-	-	-	L	L	M	ND	0	0
P23	-	-	-	-	H	H	ND	0	0
P18	-	-	-	H	H	H	ND	4	8
Group D1 sensitivity								2/12<br>**16.7%**	4/12<br>**33.3%**
Group D2 (2009)									
P26	-	-	-	M	H	H	0	0	2
P29	-	-	-	H	H	H	0	0	4
P32	-	-	-	M	M	M	0	0	1
P34	M	-	-	H	H	H	0	5	6
Group D2 sensitivity							0/4<br>**0%**	1/4<br>**25.0%**	2/4<br>5**0.0%**
Combined Group D sensitivity							0/4<br>**0%**	3/16<br>**18.8%**	6/16<br>**37.5%**
Group (A + B) sensitivity							3/6<br>**50.0%**	7/16<br>**43.8%**	12/16<br>**75.0%**
Group (A + B + C) sensitivity							3/8<br>**37.5%**	7/20<br>**35.0%**	13/20<br>**65.0%**
Group (A + B + C + D) sensitivity							3/12<br>**25.0%**	10/36<br>**28.6%**	19/36<br>**52.8%**

Results expressed as ELISA or TUBEX scores; notations as in Table S1.
