# Supplementary material for: Combined Rapid (TUBEX) Test for Typhoid-Paratyphoid A Fever Based on Strong Anti-O12 Response: Design and Critical Assessment of Sensitivity
Source: PLoS One. 2011 Sep 15;6(9):e24743. doi: 10.1371/journal.pone.0024743 (PMC3174194; doi:10.1371/journal.pone.0024743)
Supplement: Table S3 — Comparison of assay performance among various TUBEX tests in the detection of (A) purified mAb P4E8, and (B) various typhoid and paratyphoid sera. (PDF) [file pone.0024743.s003.pdf]

Table S3 Comparison of assay performance among various TUBEX tests in the detection of (A) purified mAb P4E8, and (B) various typhoid and paratyphoid sera.

A

	Inhibiting mAb (μg/ml)				
	64 μg/ml	32 μg/ml	16 μg/ml	8 μg/ml	0 μg/ml
TUBEX 12TPP	6	4	2	0	0
TUBEX 12TP	6	4	2	0	0
TUBEX 12T	6	4	2	0	0
TUBEX12P	6	4	2	0	0

B

Specimen no.	TUBEX					
	TF	PA	12T	12P	12TP	12TPP
Typhoid:						
T35a	5	6	7	6	8	ND
T35b	7	8	9	8	9	ND
T35 (a+b)	ND	ND	ND	ND	ND	9
T37a	6	2	8	5	7	ND
T37b	5	2	8	5	7	ND
T37 (a+b)	ND	ND	ND	ND	ND	6
T57a	3	0	5	1	2	ND
T57b	4	2	6	4	6	ND
T57 (a+b)	ND	ND	ND	ND	ND	4
T39b	4	8	9	9	9	9
T45a	6	8	7	7	7	7
T59b	4	2	6	6	7	7
T67a	5	4	4	5	6	6
Paratyphoid:						
P01	ND	6	ND	8	6	ND
P12	ND	1	ND	8	4	6
P19	ND	6	ND	9	ND	7
P36	0	3	ND	6	6	ND

Results expressed as TUBEX scores; (a+b) denotes equally-mixed proportions of ‘a’ and ‘b’ specimens; results in (B) for TUBEX TF, TUBEX PA, TUBEX 12T and TUBEX 12P, obtained previously (Table S1 and S2); ND, not done (insufficient specimen)
