# Supplementary material for: Combined Rapid (TUBEX) Test for Typhoid-Paratyphoid A Fever Based on Strong Anti-O12 Response: Design and Critical Assessment of Sensitivity
Source: PLoS One. 2011 Sep 15;6(9):e24743. doi: 10.1371/journal.pone.0024743 (PMC3174194; doi:10.1371/journal.pone.0024743)
Supplement: Table S4 — Effect of using double-volume specimen in TUBEX TF (TUBEX TFX) in the detection of (A) purified mAb 3h1, and (B) various typhoid and paratyphoid sera. (PDF) [file pone.0024743.s004.pdf]

Table S4 Effect of using double-volume specimen in TUBEX TF (TUBEX TFX) in the detection of (A) purified mAb 3h1, and (B) various typhoid and paratyphoid sera.

A

	Inhibiting mAb conc		
	32 µg/ml	16 µg/ml	8 µg/ml
TUBEX TF	4	2	0
TUBEX TFX	6	4	2

B

Specimen no.	TUBEX		
	TF	TFX	bTFX
Typhoid:			
T39a	3	5	4
T69a	4	6	4
T53a	2	5	4
Paratyphoid:			
#P29 + P32	ND	0	0
#P31 + P35	ND	0	0
P09	ND	0	0
P14	ND	0	0
P16	ND	2	2
P18	ND	0	0

#Equal proportion of sera mixed.

Results expressed as TUBEX scores; bTFX, soluble *S.* Paratyphi A LPS added as blocker in TUBEX TF; ND, not done (insufficient specimen).
