# Supplementary material for: Combined Rapid (TUBEX) Test for Typhoid-Paratyphoid A Fever Based on Strong Anti-O12 Response: Design and Critical Assessment of Sensitivity
Source: PLoS One. 2011 Sep 15;6(9):e24743. doi: 10.1371/journal.pone.0024743 (PMC3174194; doi:10.1371/journal.pone.0024743)
Supplement: Table S5 — Antibody activity of sera from healthy controls or non- Salmonella febrile patients determined by various ELISA and TUBEX tests. (PDF) [file pone.0024743.s005.pdf]

Table S5 Antibody activity of sera from healthy controls or non-*Salmonella* febrile patients determined by various ELISA and TUBEX tests.

Specimen no:	ELISA IgM			ELISA IgG			TUBEX			
	T-LPS	**P-LPS**	M-LPS	T-LPS	**P-LPS**	M-LPS	**TF**	PA	**12P**	12T
C1	L	-	-	-	-	-	2	0	0	1
C2	-	-	-	-	M	M	1	0	0	0
C3	L	L	-	-	-	-	3	0	1	2
C4	L	L	L	-	-	-	4	ND	0	3
C5[#]	-	-	-	-	-	-	0	0	0	0
C6[#]	-	-	-	-	-	-	0	0	0	0
C7	-	-	-	-	-	-	0	ND	ND	0
C8	L	-	L	-	-	-	3	0	0	3
C9	-	-	-	-	-	-	2	0	0	2
C10	L	-	L	-	-	-	0	0	0	0
C11	-	-	-	H	M	M	0	0	0	2
C12	-	-	-	-	-	-	0	0	0	1
C13	-	-	-	-	-	L	0	0	0	2
C14	-	-	-	-	-	-	0	0	0	0
C15	-	-	-	-	-	-	0	0	0	0
C16	-	-	-	-	-	-	0	0	0	0
C17[#]	L	L	L	-	-	-	0	0	2	0
C18	-	-	-	-	-	-	0	0	0	0
C19	-	-	-	-	-	-	0	0	0	0
C20[#]	-	-	-	L	-	-	0	0	0	0
C21	-	-	-	-	-	-	0	0	0	0
C22	-	-	L	-	-	-	1	2	2	4
C23	-	-	-	-	-	-	ND	ND	0	2
C24	-	-	-	-	-	-	0	ND	0	0
C25	-	-	L	-	-	-	2	0	2	4
C26	-	-	-	-	-	L	0	0	0	0
C27	-	-	-	-	L	-	ND	ND	0	0
C28	-	-	-	-	-	-	ND	ND	0	1
C29	-	L	L	-	-	-	0	0	0	2
C30	-	-	-	-	-	-	0	0	0	1

[#]TUBEX 12TP and TUBEX 12TPP performed subsequently yielded score ‘0’.

C1-C20, healthy healthcare workers; C21-C30, non-*Salmonella* febrile patients; other notations as in Table S1.
